# Prevalence of intestinal parasitic infections in patients with diabetes: a systematic review and meta-analysis

**DOI:** 10.1093/inthealth/ihad027

**Published:** 2023-04-13

**Authors:** Ali Taghipour, Ehsan Javanmard, Hanieh Mohammad Rahimi, Amir Abdoli, Sara Matin, Marzieh Haghbin, Meysam Olfatifar, Hamed Mirjalali, Mohammad Reza Zali

**Affiliations:** Zoonoses Research Center, Jahrom University of Medical Sciences, Jahrom 74148-46199, Iran; Department of Medical Parasitology and Mycology, School of Public Health, Tehran University of Medical Sciences, Tehran 1417613151, Iran; Foodborne and Waterborne Diseases Research Center, Research Institute for Gastroenterology and Liver Diseases, Shahid Beheshti University of Medical Sciences, Tehran 4739-19395, Iran; Zoonos es Research Center, Jahrom University of Medical Sciences, Jahrom 74148-46199, Iran; Department of Medical Parasitology and Mycology, School of Medicine, Jahrom University of Medical Sciences, Jahrom 74148-46199, Iran; Department of Pediatrics, Jahrom University of Medical Sciences, Jahrom 74148-46199, Iran; Clinical Research Development Unit of Peymanieh Hospital, Faculty of Medicine, Jahrom University of Medical Sciences, Jahrom 3713649373, Iran; Gastroenterology and Hepataology Diseases Research Center, Qom University of Medical Sciences, Qom 4739-19395, Iran; Foodborne and Waterborne Diseases Research Center, Research Institute for Gastroenterology and Liver Diseases, Shahid Beheshti University of Medical Sciences, Tehran 4739-19395, Iran; Gastroenterology and Liver Diseases Research Center, Research Institute for Gastroenterology and Liver Diseases, Shahid Beheshti University of Medical Sciences, Tehran 4739-19395, Iran

**Keywords:** diabetes, helminths, intestinal parasites infection, meta-analysis, protozoa, systematic review

## Abstract

Patients with diabetes are at an increased risk of intestinal parasitic infections (IPIs). We evaluated the pooled prevalence and OR of IPIs in patients with diabetes through a systematic review and meta-analysis. A systematic search was performed using the Preferred Reporting Items for Systematic Reviews and Meta-Analyses (PRISMA) protocol for studies reporting IPIs in patients with diabetes through 1 August 2022. The collected data were analyzed using comprehensive meta-analysis software version 2. Thirteen case-control studies and nine cross-sectional studies were included in this study. The overall prevalence of IPIs in patients with diabetes was calculated to be 24.4% (95% CI 18.8 to 31%). Considering the case-control design, the prevalence of IPIs in case (25.7%; 95% CI 18.4 to 34.5%) was higher than controls (15.5%; 95% CI 8.4 to 26.9%) and a significant correlation was observed (OR, 1.80; 95% CI 1.08 to 2.97%). Moreover, a significant correlation was seen in the prevalence of *Cryptosporidium* spp. (OR, 3.30%; 95% CI 1.86 to 5.86%), *Blastocystis* sp. (OR, 1.57%; 95% CI 1.11 to 2.22%) and hookworm (OR, 6.09%; 95% CI 1.11 to 33.41%) in the cases group. The present results revealed a higher prevalence of IPIs in patients with diabetes than in controls. Therefore, the results of this study suggest a proper health education program to preventing measures for the acquisition of IPIs in patients with diabetes.

## Background

Intestinal parasitic infections (IPIs) are still a major health problem worldwide, particularly in nations with poor sanitation status.^1,2^ It is estimated that approximately 3.5 billion people are exposed to various types of IPIs, of whom 450 million people experience a wide range of parasitic infections.^3,4^ IPIs have continued to prevail in underdeveloped countries, due to the sanitation shortage, poor hygienic conditions and neglect of health education.^5–8^ IPIs are one of the major causes of digestive disorders (such as diarrhea, nausea and vomiting), chronic malabsorption and malnutrition, and failure to thrive, especially among high-risk groups, namely, children, pregnant women and immunocompromised patients.^9–13^ Moreover, there are is evidence showing a probable linkage between autoimmune diseases and the presence/absence of some IPIs.^14–16^

There are two main types of diabetes: type 1 diabetes (T1D), which is characterized by hyperglycemia elicited by deficiencies in insulin hormone release; and type 2 diabetes, due to failure to properly respond to insulin in target cells.^17^ However, these conditions are probably caused by genetic background, immune/autoimmune reactions, environmental factors and lifestyle.^18–20^ As of 2017, it was estimated that at least 425 million people had diabetes worldwide with approximately 3.2 to 5.0 million diabetes-related deaths.^21^ Currently, there are is evidence of a probable relationship of diabetes and infectious agents like bacterial and viral infections.^22,23^ It was demonstrated that patients with diabetes are at an increased risk of herpes zoster infection.^22^ Indeed, it was suggested that Helicobacter pylori infection may lead to an increased incidence rate of diabetes.^23^ On the other hand, there are epidemiological data and experimental evidence demonstrating the role of protozoa and helminths in provoking a subset of immune responses.^24^ Actually, parasite protozoa mainly induce Th1 responses,^25,26^ while helminths induce Th2 responses and subsequent immunological cascades.^26,27^ However, numerous studies have evaluated the potential role of helminths or their products in balancing immune responses during autoimmune diseases, such that the results were promising.^28–30^ Because helminth parasites mostly attenuate inflammation by the induction of anti-inflammatory cytokines such as IL4, IL10 and TGFβ, the presence of helminths may change metabolic pathways in adipose tissue, increase insulin sensitivity and reduce the risk of T1D.^31,32^ On the other hand, concerning the inverse immunological responses in infection with the parasitic protozoa, it seems that a synergistic effect of the presence of protozoan parasites and insensitivity of insulin would be expected.^33,34^ Therefore, we accomplished a systematic review with a meta-analysis approach focusing on the prevalence and OR of IPIs in patients with diabetes.

## Methods

### Search strategy

The Preferred Reporting Items for Systematic Reviews and Meta-Analyses (PRISMA) protocols were followed for this study.^35^ Hence, a systematic search was accomplished on the related records in online databases (Scopus, Web of Science and PubMed) and search engines (and Google Scholar) from their inception until 1 August 2022. The searching procedure was conducted using combinations of the following search keywords, including: ‘Parasite,’ ‘Intestinal parasites,’ ‘Helminth,’ ‘Protozoan,’, ‘Protozoa,’ AND ‘Diabetes’ in the English language.

### Inclusion and exclusion criteria

The inclusion criteria included: (1) original research and short report papers; (2) studies with case-control and cross-sectional designs that assessed the prevalence of IPIs in patients with diabetes; (3) papers published with full text/abstracts in English; (4) papers published online up to 1 August 2022; (5) papers with information on the exact total sample size and infected samples; and (6) studies that have measured at least one type of IPI. All articles that did not meet the above criteria were excluded from the study.

### Types of participants

The studies were selected if their participants were people with diabetes, patients with type 1 diabetes mellitus or type 2 diabetes mellitus.

### Types of outcome measures

All included studies were on the prevalence of IPIs in patients with diabetes.

### Study selection and data extraction

The initial screening, eligibility and inclusion criteria for the downloaded articles were evaluated by two authors (AT and HMR). The selected papers were carefully examined, and disagreements between the reviewers were resolved by consensus. Then one author (AT) extracted the information and the others (HM and EJ) rechecked them. Moreover, the bibliographic list of all selected articles was hand-searched to find other relevant articles or their citations by searching in Google Scholar. Finally, the following data were extracted for each study: first author, country, year of publication, study design, diagnostic method for IPIs, type of diabetes, sample size and the prevalence of IPIs in case-control and cross-sectional studies.

### Quality assessment

In the present study, the Newcastle-Ottawa Scale (NOS) was employed to evaluate the quality of cross-sectional studies (low quality with score ≤3.5, moderate quality with 3.6–5.25 and high quality with 5.26–7)^36^ and case-control studies (low quality with score ≤4.5, moderate quality with 4.6–6.7 and high quality with 6.8–9).^37^ Finally, articles with scores >3.5 for cross-sectional studies and >4.5 for case-control studies were included in the study.

### Data synthesis and statistical analysis

All data were assessed by using comprehensive meta-analysis software version 2.^8,38^ Considering the association between helminths and protozoa with diabetes, an OR using the random effects model and a corresponding 95% CI were calculated for each study. Regarding the heterogeneity of studies, the *I*^2^ index was measured so that a value >50% indicated a statistically significant heterogeneity. Egger's regression was performed to calculate the possibility of publication bias.^39^ p<0.05 was considered statistically substantial.

## Results

### Study characteristics

**Figure 1.**
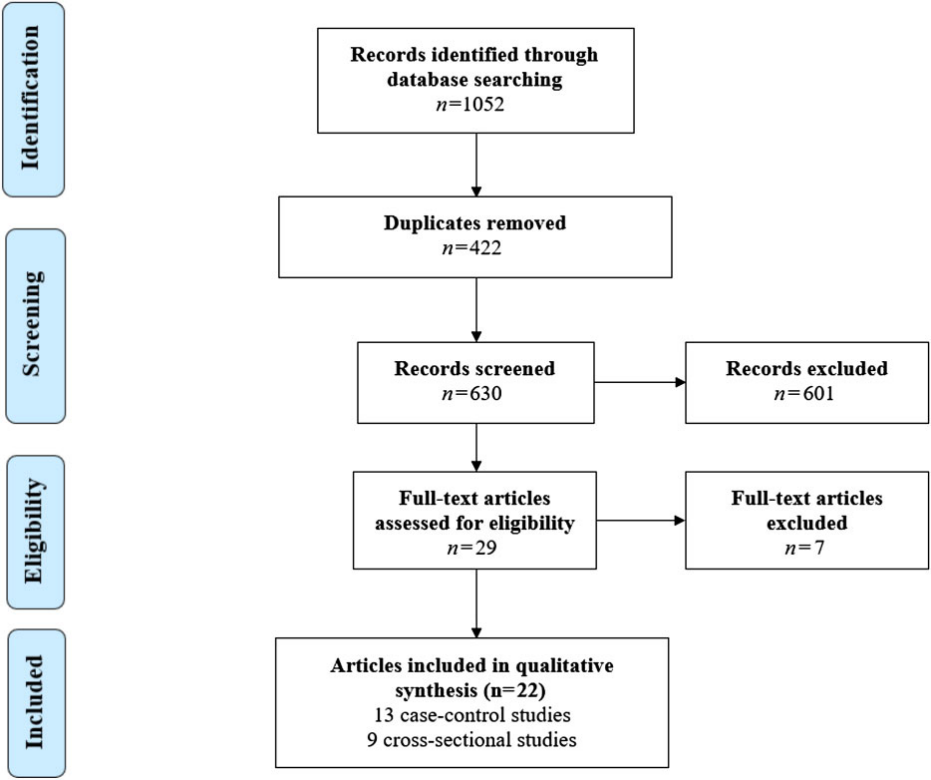
Flow diagram of the study design process.

A total of 1052 records were recognized by the primary search in the online databases (Figure 1). Then, after eliminating duplicates and papers with non-related topics, 22 articles (13 case-control^16,40–51^ and nine cross-sectional^52–60^ studies) were eligible to include in the data synthesis. The quality assessment according to the NOS for case-control and cross-sectional studies is represented in Tables 1 and 2. All included articles exhibited appropriate quality. These studies were conducted in 12 different countries (four in Egypt, three in Iran, two in Brazil, two in Ethiopia, two in Nigeria, two in Thailand, two in Turkey, one in Cameroon, one in Ghana, one in India, one in Iraq and one in Sudan). Further data are shown in Tables 1 and 2.

**Table 1.** Summary of studies with a case-control design investigating the prevalence of IPIs in diabetes patients and control subjects

First author,		Type of	Type of	Detection	Total	Total parasites	Total	Total parasites	Significant	
publication year	Country	diabetes	parasites	methods	cases	(type of parasite (n))	controls	(type of parasite (n))	difference	QA
Nazligol et al., 2001	Turkey	Mixed/unknown DM	Intestinal parasites	Lugol and flotation methods	200	**94** (*Ascaris lumbricoides* (54), *Trichuris trichiura* (13), *Entamoeba histolytica/dispar* (13), *Giardia lamblia* (11), *Taenia* spp. (3))	1024	**564** (not reported)	Yes (protective)	9
Akhlaghi et al., 2006	Iran	Mixed/unknown DM	Intestinal parasites	Formol-ether concentration and modified Ziehl–Neelsen staining	250	**39** (*G. lamblia* (9), *Entamoeba coli* (9), *Cryptosporidium* spp. (6), *Blastocystis* sp. (6), *Iodamoeba butschlii* (2), *A. lumbricoides* (2), *Hymenolepis nana* (2), 4 nucleited cyst (1), *Endolimax nana* (1), *Trichomonas hominis* (1))	250	**25** (*E. coli* (9), *G. lamblia* (7), *Blastocystis* sp. (4), *Cryptosporidium* spp. (1), *I. butschlii* (1), *A. lumbricoides* (1), *H. nana* (1), *E. nana* (1), *T. hominis* (1))	No	9
Akinbo et al., 2013	Nigeria	150 diabetic (18 type 1 and 132 type 2)	Intestinal parasites	Formol-ether concentration	150	**28= 2 cases in type 1 DM and 26 cases in type 2 DM** (hookworms (15), *A. lumbricoides* (10), E. histolytica/dispar (3))	30	**0**	Yes	7
Mohtashamipour et al., 2015	Iran	Mixed/unknown DM	Intestinal parasites	Formol-ether concentration, Kinyoun acid-fast staining, modified trichrome stain (ryan-blue)	118	**31** (*Blastocystis* sp. (11), *E. nana* (6), *G. lamblia* (4), *E. coli* (3), *Cryptosporidium* spp. (2), *I. butschlii* (2), *Enterobius vermicularis* (2), microsporidia (2), *H. nana* (1), *T. hominis* (1), *Chilomastix mesnili* (1))	118	**8** (*E. nana* (4), *Blastocystis* sp. (3), *G. lamblia* (1), *E. coli* (1), *I. butschlii* (1))	Yes	8
Tangi et al., 2016	Cameroon	Mixed/unknown DM	Intestinal parasites	Formol-ether concentration and Ziehl–Neelsen staining	150	**15** (*E. histolytica/dispar* (10), *Blastocystis* sp. (4), *A. lumbricoides* (1), hookworms (1), *Cryptosporidium* spp. (1))	85	**20** (*E. histolytica/dispar* (18), *A. lumbricoides* (2), *Blastocystis* sp. (1))	Yes (protective)	8
Poorkhosravani et al., 2019	Iran	Mixed/unknown DM	Intestinal parasites	Direct smear, concentration method, acid-fast and trichrome staining, agar plate cultivation, Baermann technique	254	**32** (*Blastocystis* sp. (23), *E. nana* (10), *Strongyloides stercoralis* (6), *E. coli* (5), *G. lamblia* (3))	247	**46** (*Blastocystis* sp. (25), *E. coli* (12), *E. nana* (10), *G. lamblia* (8), *S. stercoralis* (4), *I. bustchlii* (2), *C. mesnilli* (1), *Entamoeba hartmani* (1))	No	9
Rady et al., 2019	Egypt	T1DM	Intestinal parasites	Lugol staining, formol-ether concentration and modified Ziehl–Neelsen staining	175	**71** (*G. lamblia* (18), *Cryptosporidium* spp. (17), *Blastocystis* sp. (16), *E. histolytica/dispar* (13), *H. nana* (6), *A. lumbricoides* (1))	238	**30** (*Blastocystis* sp. (9), *E. histolytica/dispar* (7), *A. lumbricoides* (4), *H. nana* (4), *G. lamblia* (4), *Cryptosporidium* spp. (2))	Yes	9
Popruk et al., 2020	Thailand	Mixed/unknown DM	*Blastocystis* sp.	PCR	130	**16** (not reported)	100	**9** (not reported)	No	6
Fadl et al., 2021	Egypt	DM	Intestinal protozoa	Direct smear and formalinethyl acetate sedimentation methods	40	**11** (*Blastocystis* sp. (10), *Cryptosporidium* spp. (6))	40	**5** (*Blastocystis* sp. (2), *Cryptosporidium* spp. (2), *E. histolytica/dispar* (1))	No	6
de Melo et al., 2021	Brazil	T2DM	*Blastocystis* sp.	PCR	99	**34** (not reported)	76	**23** (not reported)	No	7
Maori et al., 2021	Nigeria	Mixed/unknown DM	Intestinal parasites	Direct smear and formalin-ether concentration techniques	138	**70** (*S. stercoralis* (41), hookworms (18), *E. histolytica/dispar* (7), *S. stercoralis*+hookworms (2), *E. histolytica/dispar*+hookworms (2))	46	**4** (*E. histolytica/dispar* (3), *G. lamblia* (1))	Yes	6
Almugadam et al., 2021	Sudan	T2DM	Intestinal parasites	Simple-direct saline method, formol-ether concentration technique and modified Ziehl–Neelsen method	150	**31** (*Cryptosporidium* spp. (11), *E. histolytica/dispar* (6), *H. nana* (6), *Schistosoma mansoni* (5), *Cryptosporidium* spp.+*E. histolytica* (3))	150	**16** (*Cryptosporidium* spp. (6), *H. nana* (4), *S. mansoni* (3), *E. histolytica/dispar* (3))	Yes	7
Waly et al., 2021	Egypt	Mixed/unknown DM	Intestinal parasites	Direct smear, concentration technique, permanent staining by modified Ziehl–Neelsen and modified trichrome stains, and culture on nutrient agar plates	100	**44** (*Blastocystis* sp. (29), *Cryptosporidium* spp. (12), *G. lamblia* (8), microsporidia (5), *E. histolytica/dispar* (2), *H. nana* (2), *Capillaria philippinensis* (2))	100	**32** (*Blastocystis* sp. (21), *G. lamblia* (7), *Cryptosporidium* spp. (5), *H. nana* (4), *E. histolytica/dispar* (2), *C. philippinensis* (1))	No	7

Abbreviations: DM, diabetes mellitus; QA, quality assessment; T1DM, type 1 diabetes mellitus; T2DM, type 2 diabetes mellitus.

**Table 2.** Summary of studies with a cross-sectional design investigating the prevalence of IPIs in diabetes patients

First author, publication year	Country	Type of diabetes	Type of parasites	Detection methods	No. in diabetes group	Total parasite (parasite type (n))	QA
Machado et al., 2018	Brazil	156 diabetic (120 type 1 and 36 type 2)	Intestinal parasites	Formol-ether concentration and Ziehl–Neelsen staining	156	**102=74 cases in type 1 DM and 28 cases in type 2 DM** (*Entamoeba coli* (65), *Endolimax nana* (36), *Giardia lamblia* (25), *Ascaris lumbricoides* (19), *Entamoeba hartmanni* (16), *Taenia* spp. (5), hookworm (3), *Entamoeba histolytica/dispar* (2), *Balantidium coli* (1), *Hymenolepis nana* (2), *Strongyloides stercoralis* (1), *Enterobius vermicularis* (1), *Schistosoma mansoni* (1), mixed parasites (49))	**7**
Alemu et al., 2018	Ethiopia	DM	Intestinal parasites	Direct smear, formol-ether concentration and modified Ziehl–Neelson staining techniques	215	**42** (*Cryptosporidium* spp. (18), *A. lumbricoides* (8), *G. lamblia* (6), hookworm (4), *Trichuris trichiura* (4), *Taenia* spp. (2))	**7**
El Drawany et al., 2019	Egypt	T1DM	Intestinal parasites	Direct smear, iodine stained smears, formol-ether concentration and modified Ziehl–Neelsen staining	185	**50** (*Cryptosporidium* spp. (16), *Blastocystis* sp. (10), *G. lamblia* (4), *H. nana* (4), *S. stercoralis* (3), *A. lumbricoides* (2), *T. trichuria* (2), *E. histolytica/dispar* (2), mixed parasites (7))	**7**
Chandi et al., 2020	India	DM	Intestinal parasites	Concentration and modified Ziehl–Neelsen staining techniques	110	**15** (*E. histolytica/dispar* (8), *Cryptosporidium parvum* (5), *A. lumbricoides* (2), *G. lamblia* (1))	**7**
Ambachew et al., 2020	Ethiopia	DM	Intestinal parasites	Direct smear and formal ether concentration technique	234	**45** (*A. lumbricoides* (15), *E. histolytica/dispar* (9), hookworm (9), *S. mansoni* (7), *E. vermicularis* (3), *G. lamblia* (2))	**7**
Al-Mousawi and Nemah, 2021	Iraq	DM	Intestinal parasites	Lugols-Iodine and Ziehl–Neelsen staining methods	372	**137** (*E. histolytica/dispar* (47), *G. lamblia* (39), *A. lumbricoides* (19), *C. parvum* (9), unknown (23))	**7**
Sisu et al., 2021	Ghana	DM	Intestinal parasites	Direct smear, formol-ether concentration, and modified Ziehl–Neelsen staining methods	152	**19** (*G. lamblia* (9), *E. histolytica/dispar* (4), *C. parvum* (3), *E. coli* (3), *A. lumbricoides* (1), hookworm (1))	**7**
Yingklang et al., 2022	Thailand	T2DM	*Strongyloides stercoralis*	Modified agar plate culture and the formalin-ethyl acetate concentration technique	283	**32 (not reported)**	6
Yilmaz, 2022	Turkey	T2DM	Intestinal protozoa	Rapid antigen test	65	**11** (*C. parvum* (3), *G. lamblia* (2) and *G. lamblia* + *E. histolytica* (6))	**6**

Abbreviations: DM, diabetes mellitus; QA, quality assessment; T1DM, type 1 diabetes mellitus; T2DM, type 2 diabetes mellitus.

### The pooled prevalence of IPIs in patients with diabetes

**Figure 2.**
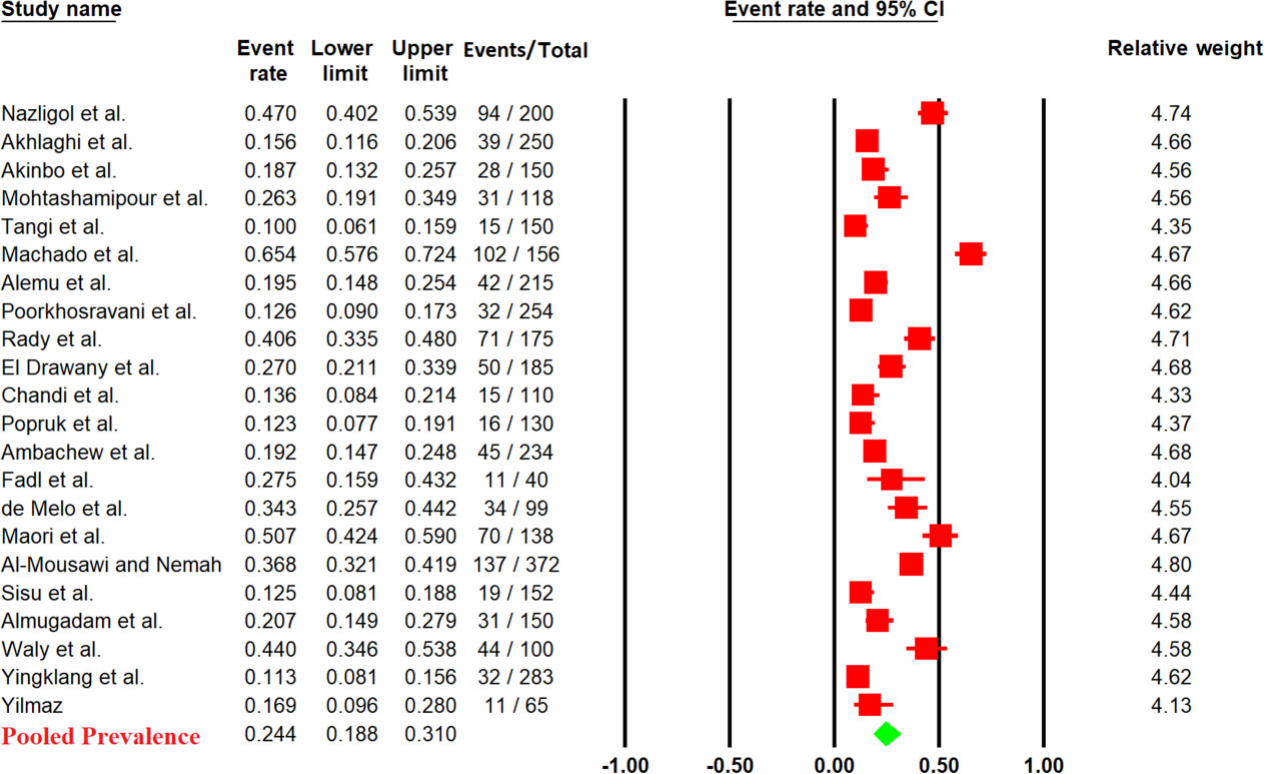
Forest plot of pooled prevalence of IPIs in diabetes patients.

As shown in Figure 2, the pooled prevalence of IPIs in patients with diabetes was calculated to be (24.4%; 95% CI 18.8 to 31%). The heterogeneity was substantial (*I*^2^=94.34%). Furthermore, subgroup analysis of pooled prevalence of IPIs in type 1 diabetes mellitus, type 2 diabetes mellitus and mixed/unknown diabetes mellitus was conducted (36.4%; 95% CI 21.1 to 55.2%), (26.7%; 95% CI 15.3 to 42.3%) and (22.8%; 95% CI 16.4 to 30.6%) in patients with diabetes, respectively (Supplementary Figure 1).

### The pooled OR of IPIs in case-control studies

**Figure 3.**
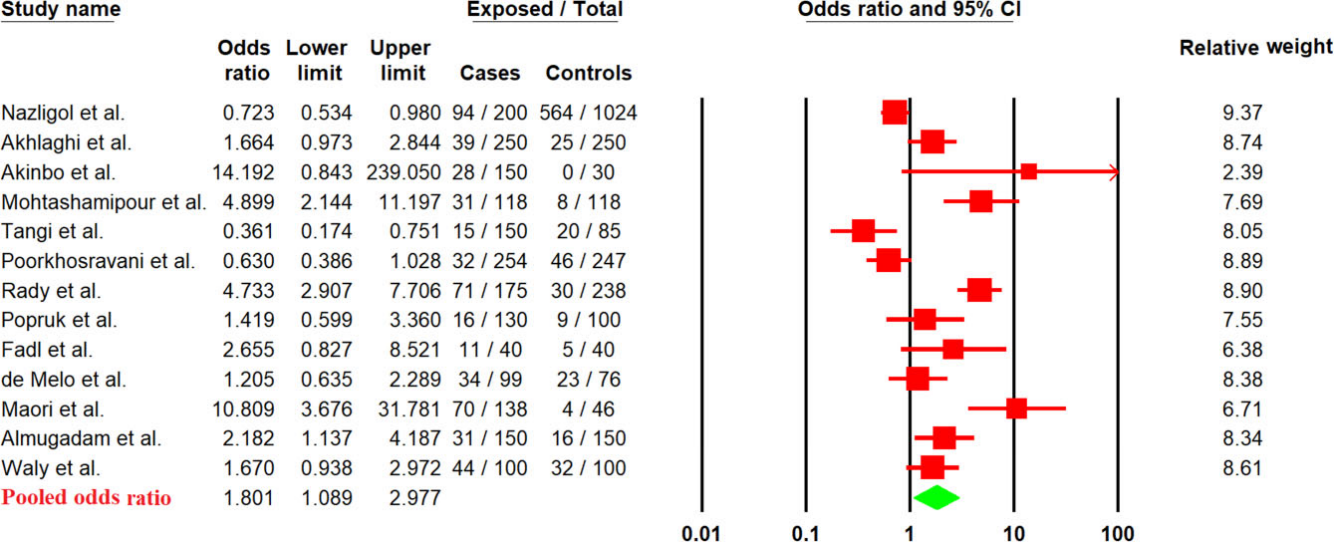
Forest plot of pooled ORs of IPIs in case-control studies.

Considering the 13 case-control studies, the pooled prevalence of IPIs in cases (25.7%; 95% CI 18.4 to 34.5%) was higher than in controls (15.5%; 95% CI 8.4 to 26.9%) (Supplementary Figures 2 and 3); a significant correlation was observed between the case and the control groups (OR, 1.80; 95% 95% CI 1.08 to 2.97%) (Figure 3). In a subgroup analysis by parasite species (Table 3), a significant association was seen between the prevalence of *Cryptosporidium* spp. (OR, 3.30%; 95% 95% CI 1.86 to 5.86%) (Supplementary Figure 4), *Blastocystis* sp. (OR, 1.57%; 95% CI 1.11 to 2.22%) (Supplementary Figure 5) and hookworm (OR, 6.09%; 95% CI 1.11 to 33.41%) (Supplementary Figure 6) between the case and control groups. Further data are provided in Table 3 and Supplementary Figures 4–13.

**Table 3.** Subgroup meta-analysis of the prevalence/OR of IPIs in diabetes patients based on case-control studies

		Cases		Controls			
Parasites	No. of studies	Infected/N	Pooled prevalence (95% CI)	Infected/N	Pooled prevalence (95% CI)	OR (95% CI)	OR heterogeneity *I*^2^ %
Protozoa							
*Blastocystis* sp.	9	149/1316	11.5 (6.4 to 19.7)	97/1254	6.7 (3.4 to 13.1)	1.57 (1.11 to 2.22)*	23.40
*Giardia lamblia*	5	42/897	4.6 (2.3 to 9)	27/953	3 (1.7 to 5.3)	1.62 (0.60 to 4.37)	68.13
*Entamoeba histolytica/dispar*	6	41/863	4.9 (3.3 to 7.2)	33/649	4.3 (1.4 to 12.4)	1.01 (0.38 to 2.64)	66.37
*Cryptosporidium* spp.	7	55/ 983	5.9 (3.2 to 10.5)	16/981	2 (0.9 to 4.4)	3.30 (1.86 to 5.86)*	0
*Entamoeba coli*	3	17/622	2.8 (1.8 to 4.5)	22/615	3.7 (2.1 to 6.5)	0.79 (0.31 to 2.01)	38.39
*Endolimax nana*	3	17/622	3 (1.2 to 7.5)	15/615	2.6 (1 to 6.5)	1.11 (0.54 to 2.27)	0
Helminths							
*Ascaris lumbricoides*	4	14/725	1.4 (0.3 to 6.3)	7/603	1.5 (0.7 to 3.1)	0.82 (0.22 to 2.97)	9.32
*Hymenolepis nana*	5	17/793	2.4 (1.3 to 4.3)	13/856	1.9 (0.9 to 3.9)	1.45 (0.69 to 3.05)	0
Hookworm	3	34/438	7.6 (3.2 to 17.2)	0	0	6.09 (1.11 to 33.41)*	0
*Strongyloides stercoralis*	2	47/392	9.4 (0.6 to 63.1)	4/293	1.5 (0.6 to 3.8)	5.95 (0.24 to 144.72)	77.12

* A significant association

### The pooled prevalence of IPIs based on the type of parasites in cross-sectional studies

Regarding the type of parasites (Table 4), the estimations of the pooled prevalence of *Entamoeba coli* (11.1%; 95% CI 0.4 to 80.5%) (Supplementary Figure 14) and *Cryptosporidium* spp. (4.8%; 95% CI 2.8 to 8%) (Supplementary Figure 15) were high among patients with diabetes. Further data are presented in Table 4 and Supplementary Figures 14–21.

**Table 4.** Subgroup meta-analysis of the prevalence of IPIs in diabetes patients based on cross-sectional studies

Parasites	No. of studies	Infected/N	Pooled prevalence (N/%)	Heterogeneity *I*^2^%
Protozoa				
*Giardia lamblia*	8	88/1489	4.2 (2.1 to 8)	85.67
*Entamoeba histolytica/dispar*	7	78/1274	4.5 (2.2 to 8.7)	83.77
*Cryptosporidium* spp.	6	54/1099	4.8 (2.8 to 8)	70.05
*Entamoeba coli*	2	68/308	11.1 (0.4 to 80.5)	97.12
Helminths				
*Ascaris lumbricoides*	7	66/1424	4.1 (2.3 to 7.2)	77.63
*Hymenolepis nana*	2	6/341	1.8 (0.8 to 4)	0
Hookworm	4	17/757	2.4 (1.3 to 4.2)	26.55
*Strongyloides stercoralis*	3	36/624	2.7 (0.4 to 15.5)	89.29

### Assessment of publication bias

Detection of publication bias using Eggers regression revealed that publication bias in case-control (p=0.08) and cross-sectional (p=0.21) studies was not statistically significant.

## Discussion

Recently, many studies have been conducted to assess the prevalence of IPIs in patients with diabetes. This meta-analysis is the first study that brings together the available information regarding the status of IPIs in patients with diabetes. Thus, our findings could be helpful for clinicians and public health policymakers, especially in countries that are endemic to both diseases.

The current review showed that the report of helminthic infections was lower than protozoa infections, and that these results were in line with the hypothesis concerning the association of IPIs (especially helminths) with diabetes explaining the beneficial role of helminths against metabolic syndrome.^61,62^

T1D is considered an autoimmune disease, during which cells of the immune system destroy the insulin-producing pancreatic β cells.^63,64^ Some evidence has shown that genetic and environmental factors may play a positive role in T1D risk.^65,66^ Thereby, it is difficult to diagnose the main cause of the disease. Based on ‘hygiene hypothesis’, epidemiological evidence suggests a steady increase in the incidence of autoimmune diseases in developed countries, coupled with a reduction in the spread of infectious diseases, particularly helminth parasites.^67^ Therefore, it can be suggested that the low prevalence of helminthic infections in recent years has increased the incidence of autoimmune diseases, especially in developed countries.^68,69^ Moreover, according to the WHO, the incidence rates of autoimmune diseases, such as T1D, in most populations in Africa and Asia, are significantly lower than in developed areas.^70,71^ Consequently, due to the small number and limited geographical distribution studies for intestinal parasites in patients with diabetes, it is necessary to conduct more accurate and quality studies to determine whether the association between intestinal parasites and diabetes is consistent with the hygiene hypothesis or vice versa.

There is evidence suggesting an interesting role for helminths in the treatment of autoimmune diseases.^28,72^ Likewise, helminths or their products have been suggested as an immunoregulator in humans. Over the years, at least 20 possible mechanisms of immunoregulatory roles of helminths have been recognized, of which regulatory T and B cells are mostly highlighted.^28,73^ Hence, intestinal helminths are able to strengthen the type 2 cell immune response (Th2) via the induction of cytokines (e.g. IL-4, IL-5, IL-9, IL-10 and IL-13).^74,75^ Accordingly, the change from Th1 toward Th2 during helminthic infections can downregulate the Th1 immune response and prevent the destruction of insulin-producing beta cells during the development of T1D.^71^

Recently, some experimental studies demonstrated that infections with helminths, including *Shistosoma mansoni* eggs, filarial proteins, *Heligmosomoides polygyrus* and excretory/secretory products from *Fasciola hepatica* in mice models, were correlated with induction of type 2 immune responses and prevention of T1D development.^71^ Nevertheless, the exact protective mechanisms of these helminths are not completely understood and might differ regarding the genus/species of parasites. Therefore, further studies are needed to understand these relationships.

Over the last decade, the role of protozoan parasites in autoimmune diseases has been evaluated. Several studies have proposed a synergistic role for *Toxoplasma* in the induction of inflammatory responses during inflammatory bowel diseases.^76–78^ However, controversial evidence suggests that *Giardia duodenalis, Blastocystis* sp. and *Dientamoeba fragilis* infections may play a role in the development of autoimmune and allergic diseases, such as irritable bowel syndrome (IBS), urticaria and skin allergies.^79–81^ In this regard, these studies mentioned that microbiota modifications in IBS patients are related to these intestinal protozoa. For example, microbiota are considered as protective bacteria due to their anti-inflammatory, anti-carcinogenic and immunostimulatory properties, and their decrease in *Blastocystis*-positive individuals may suggest a role for *Blastocystis* sp. in inflammatory disorders of the gut.^82,83^ However, studies of intestinal protozoa in patients with diabetes have been generally neglected and less considered. It is suggested that in future studies, appropriate case-control and experimental studies should be performed regarding intestinal protozoa to provide a deep understanding of the relationship between diabetes and intestinal protozoa.

This study also has some limitations so the results should be interpreted with caution: (1) a low number of epidemiological studies of IPIs in patients with diabetes worldwide; (2) some studies about the genus and species of intestinal parasites could not be extracted and analyzed; (3) most of the included studies, risk and related factors could not be evaluated; (4) a lack of evaluating the frequency of IPIs based on the type of diabetes (type 1 or 2) in some studies; and (5) a lack of online registration (PROSPERO) was another limitation to this study, because the data were already extracted (we pre-extracted the data and were unable to register it according to PROSPERO). However, there have been no similar studies in the PROSPERO database since the search was performed.

## Conclusion

A high prevalence of IPIs among patients with diabetes compared with healthy individuals was demonstrated in this meta-analysis. Hence, patients with diabetes as a high-risk group should be considered a priority for the screening of IPIs. IPIs are mainly transmitted via the fecal oral route, by consumption of contaminated water, food and raw vegetables with helminth eggs or protozoa cysts and oocysts. Therefore, patients with diabetes should be considered for preventative measures regarding IPIs. As such, planning health education programs could improve the knowledge of diabetes patients regarding transmission routes and the prevention of IPIs.

## Supplementary Material

ihad027_Supplemental_File

## Data Availability

All data gathered during the study are included in this manuscript.
