# Supplementary material for: Prevalence of intestinal parasitic infections in patients with diabetes: a systematic review and meta-analysis
Source: Int Health. 2023 Apr 13;16(1):23–34. doi: 10.1093/inthealth/ihad027 (PMC10759288; doi:10.1093/inthealth/ihad027)
Supplement: ihad027_Supplemental_File [file ihad027_supplemental_file.docx]

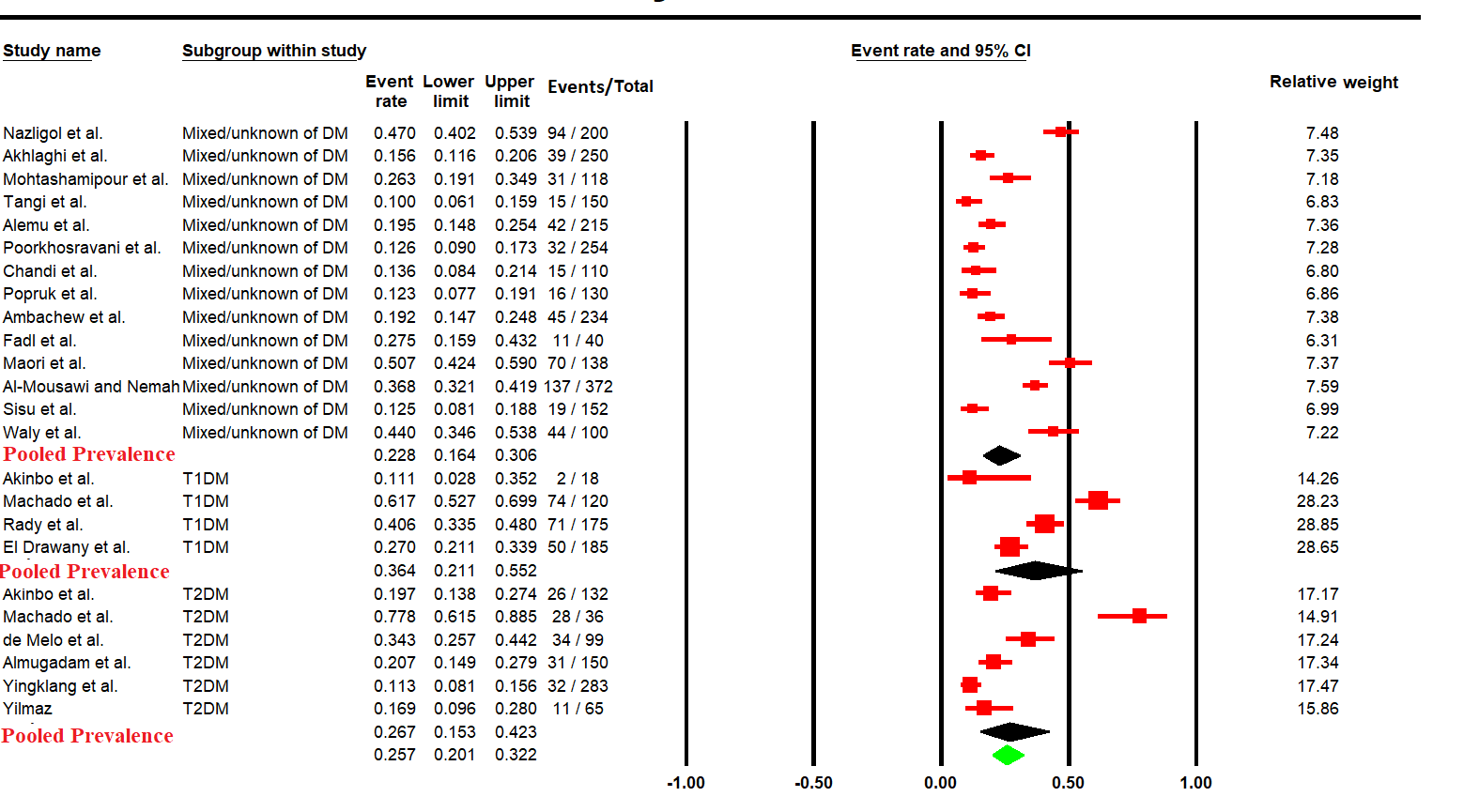


**Supplementary Figure 1.** Subgroup analysis of pooled prevalence of IPIs in type 1 diabetes (T1DM) mellitus, type 2 diabetes mellitus (T2DM), and mixed/unknown of diabetes mellitus.


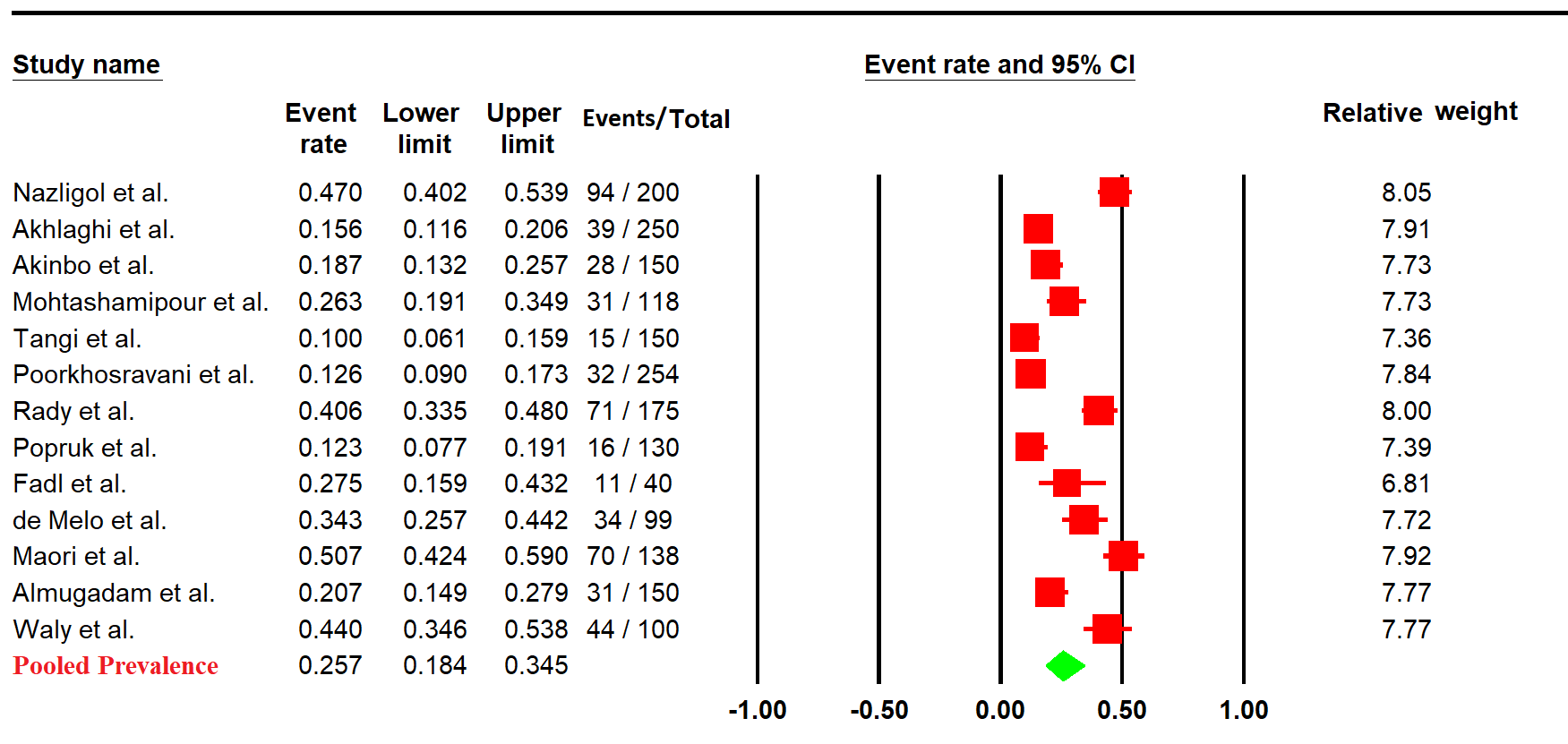


**Supplementary Figure 2.** Pooled prevalence of IPIs in diabetic patients in case-control studies.


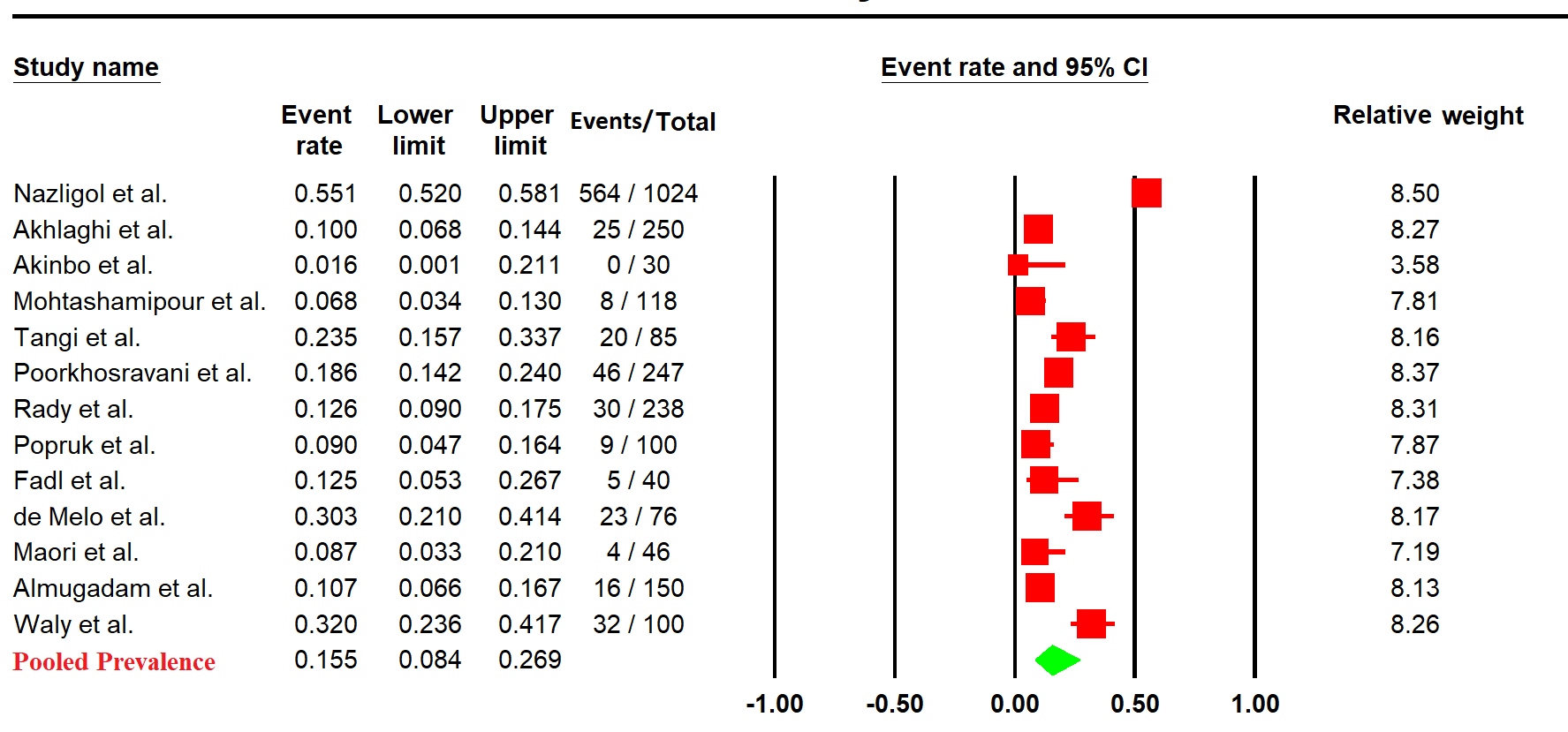


**Supplementary Figure 3.** Pooled prevalence of IPIs in control group in case-control studies.


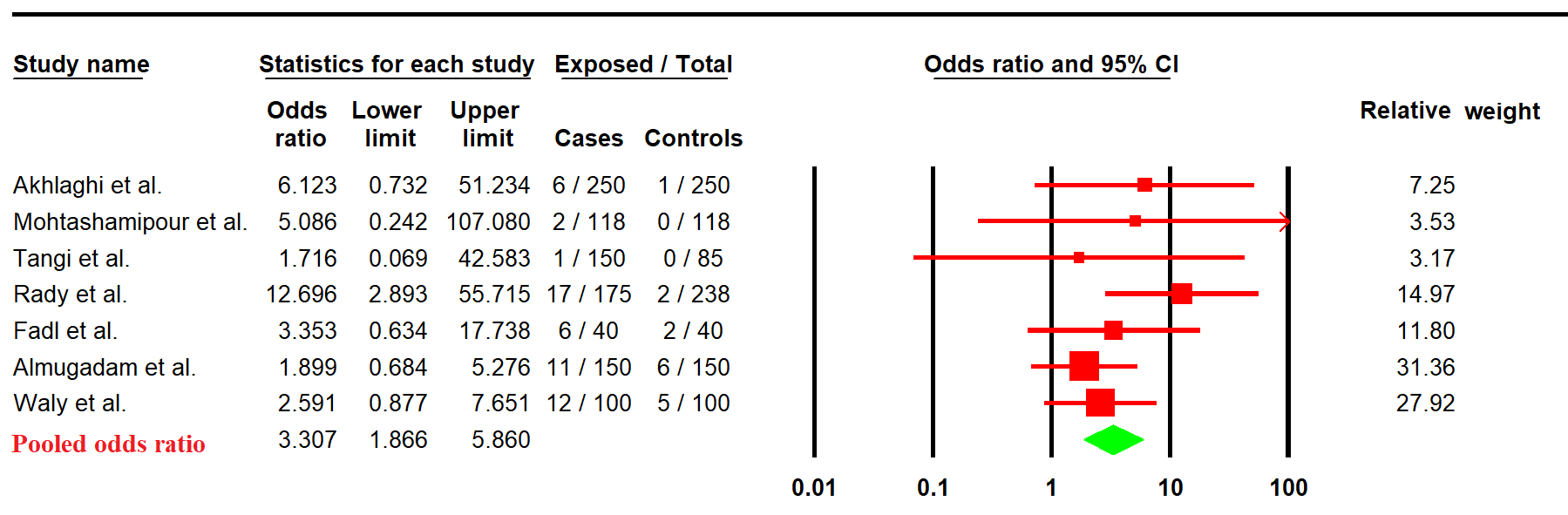


**Supplementary Figure 4.** Pooled odds ratio of *Cryptosporidium* spp. in case-control studies.


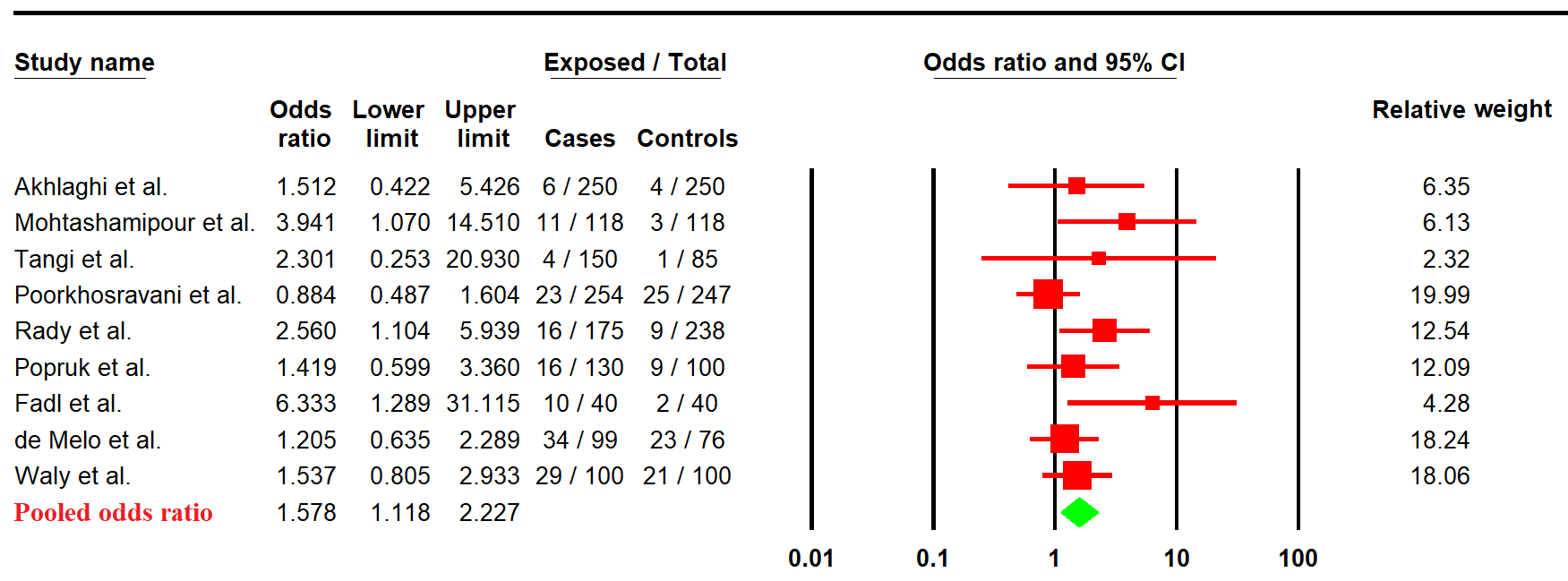


**Supplementary Figure 5.** Pooled odds ratio of *Blastocystis* sp. in case-control studies.


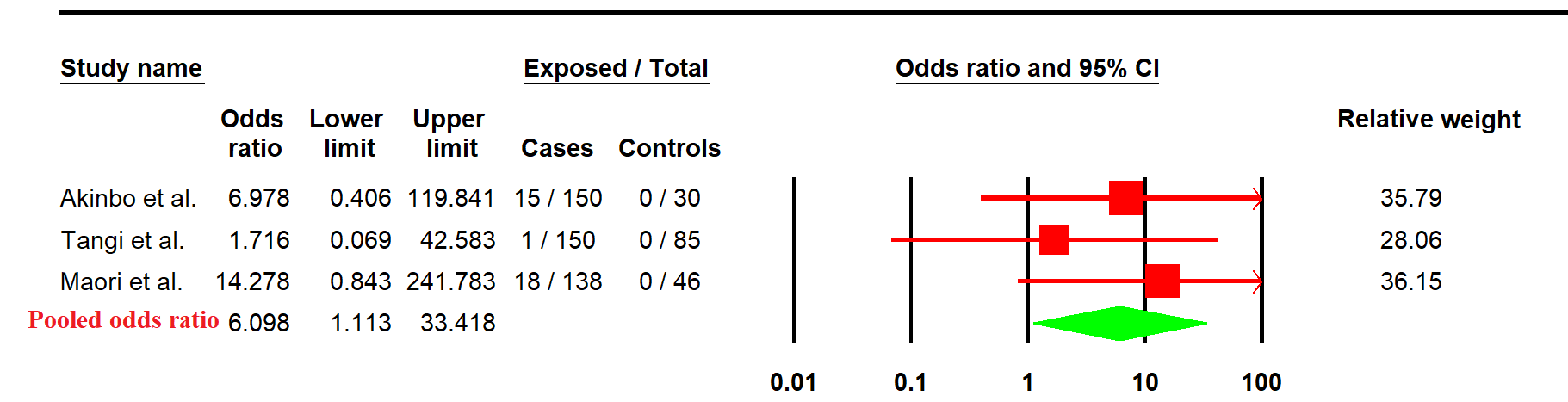


**Supplementary Figure 6.** Pooled odds ratio of Hookworm in case-control studies.


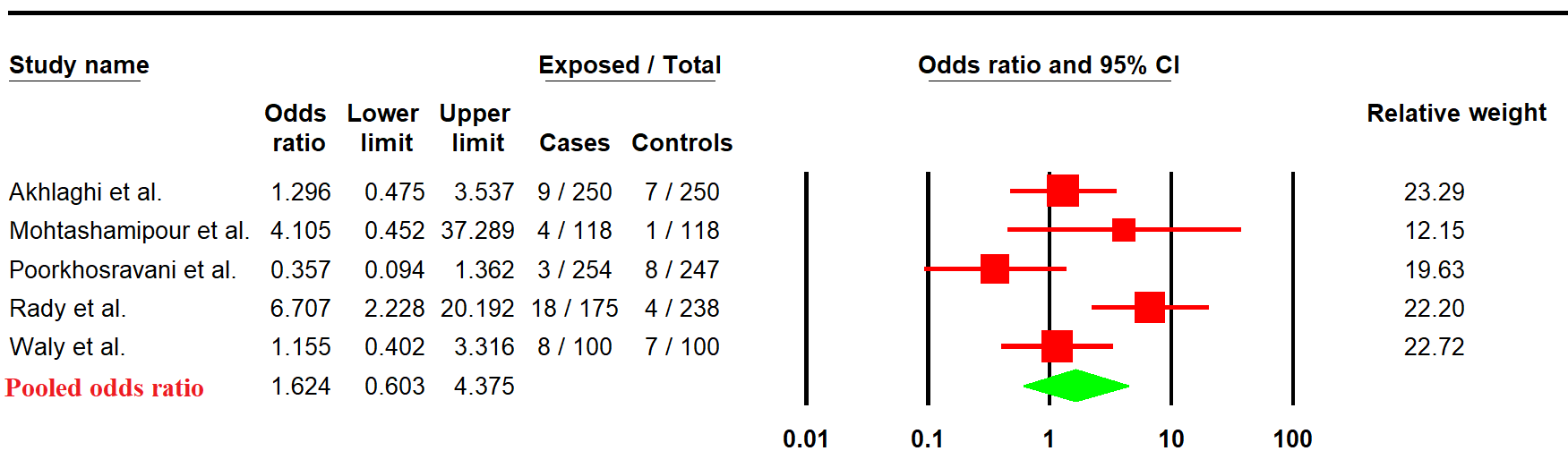


**Supplementary Figure 7.** Pooled odds ratio of *Giardia* spp. in case-control studies.


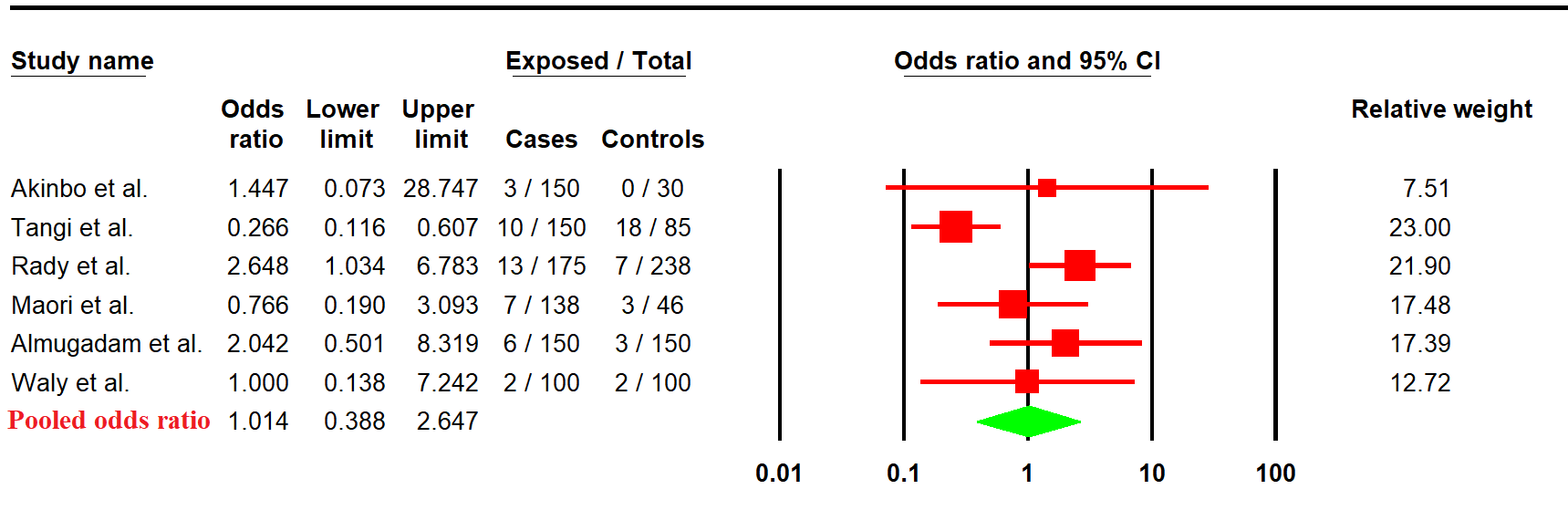


**Supplementary Figure 8.** Pooled odds ratio of *Entamoeba histolytica/dispar* in case-control studies.


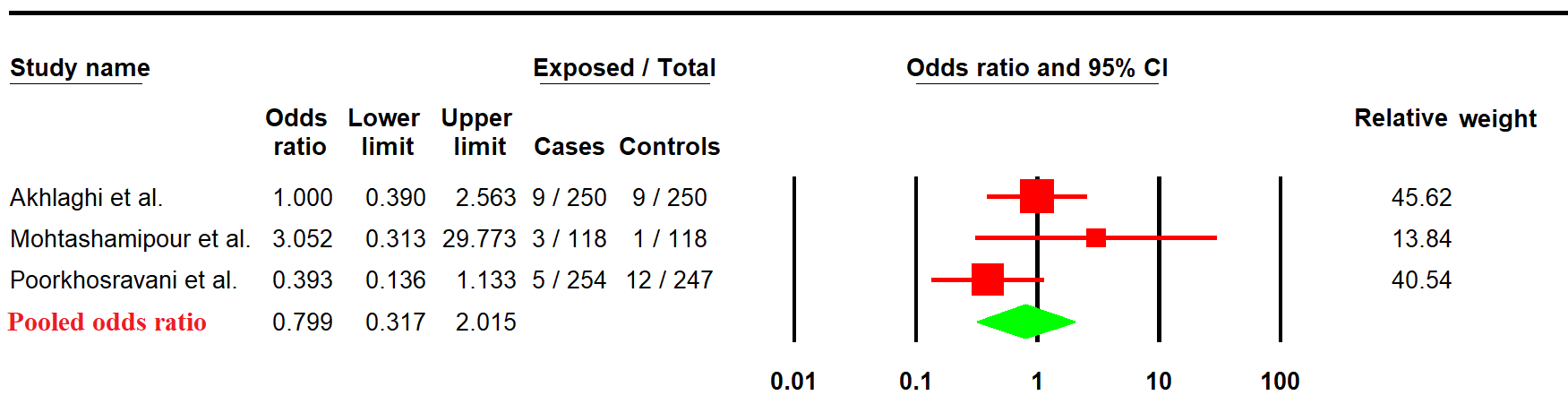


**Supplementary Figure 9.** Pooled odds ratio of *Entamoeba coli* in case-control studies.


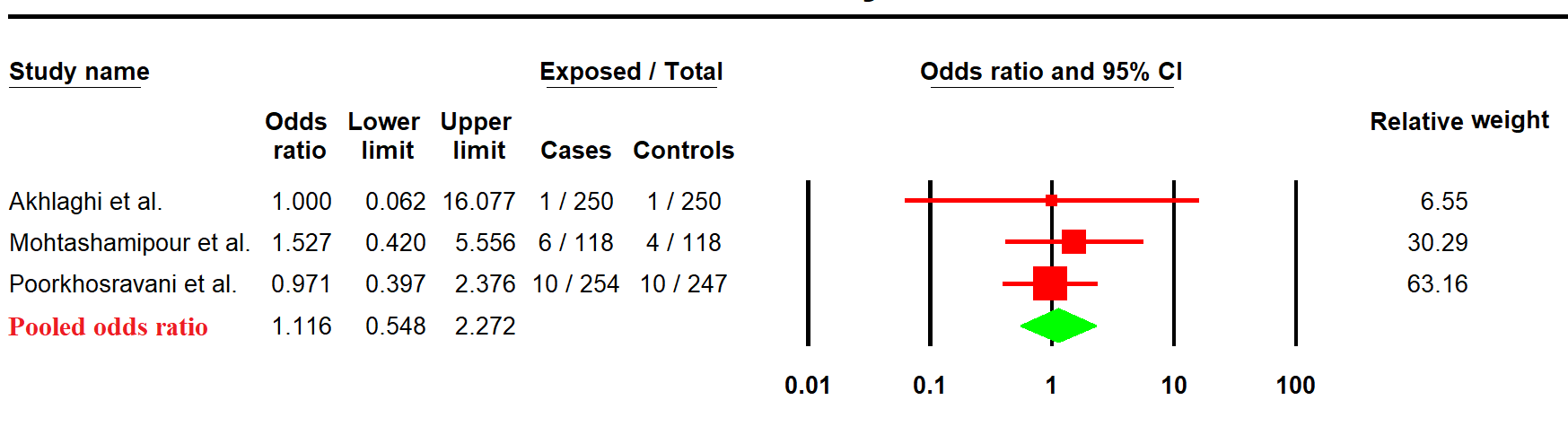


**Supplementary Figure 10.** Pooled odds ratio of *Endolimax nana* in case-control studies.


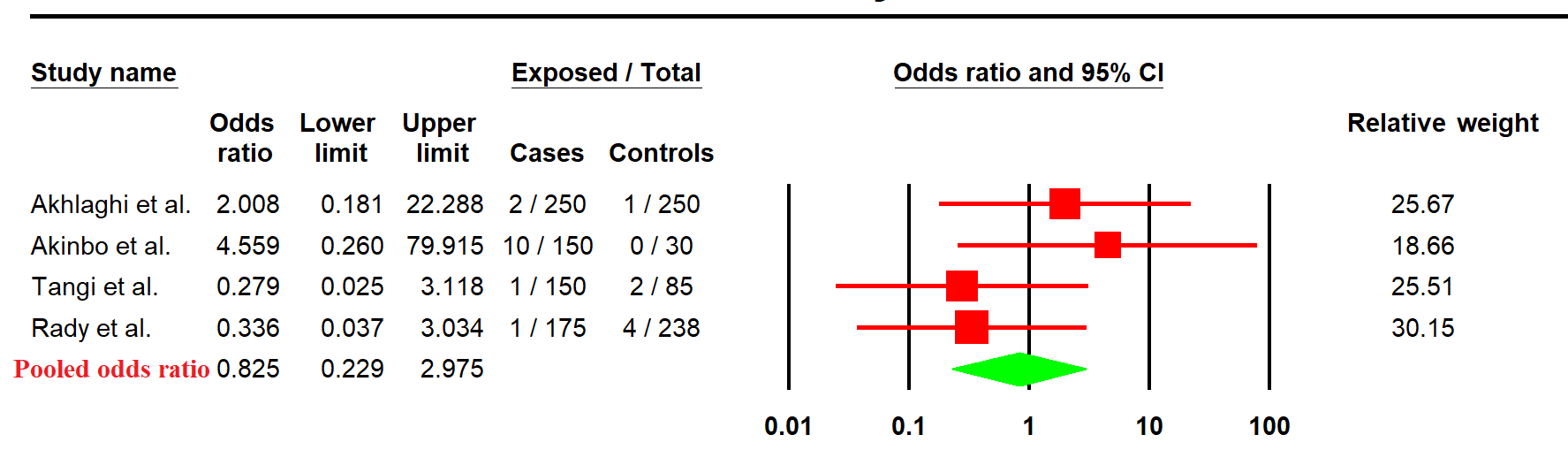


**Supplementary Figure 11.** Pooled odds ratio of *Ascaris lumbricoides* in case-control studies.


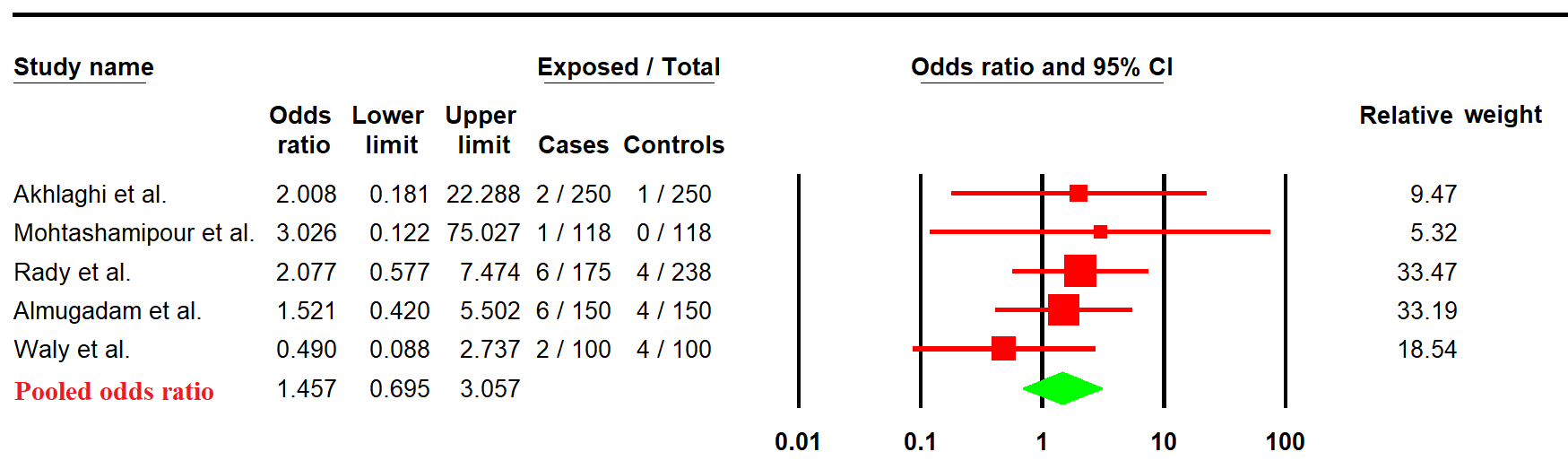


**Supplementary Figure 12.** Pooled odds ratio of *Hymenolepis nana* in case-control studies.


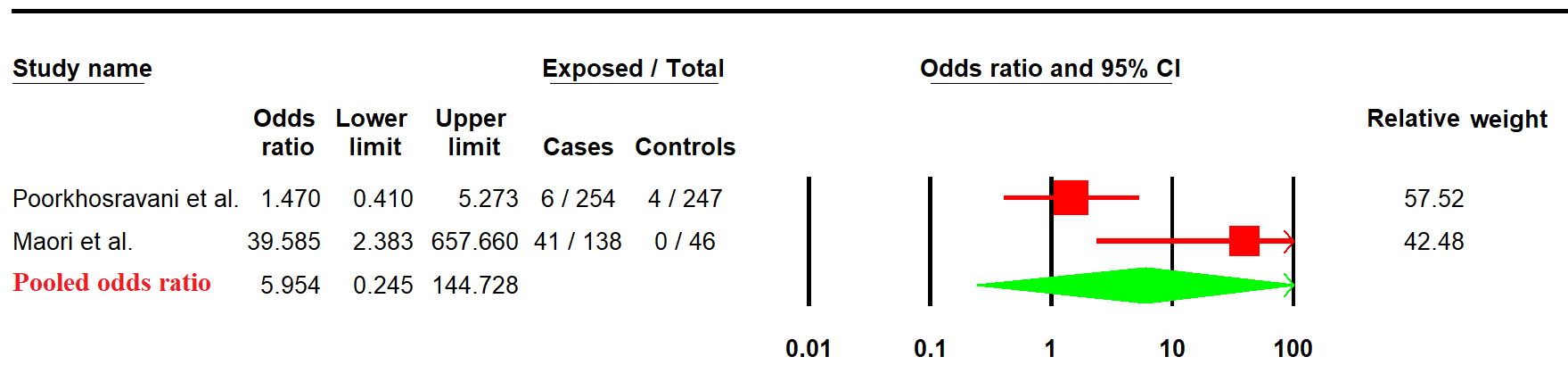


**Supplementary Figure 13.** Pooled odds ratio of *Strongyloides stercoralis* in case-control studies.


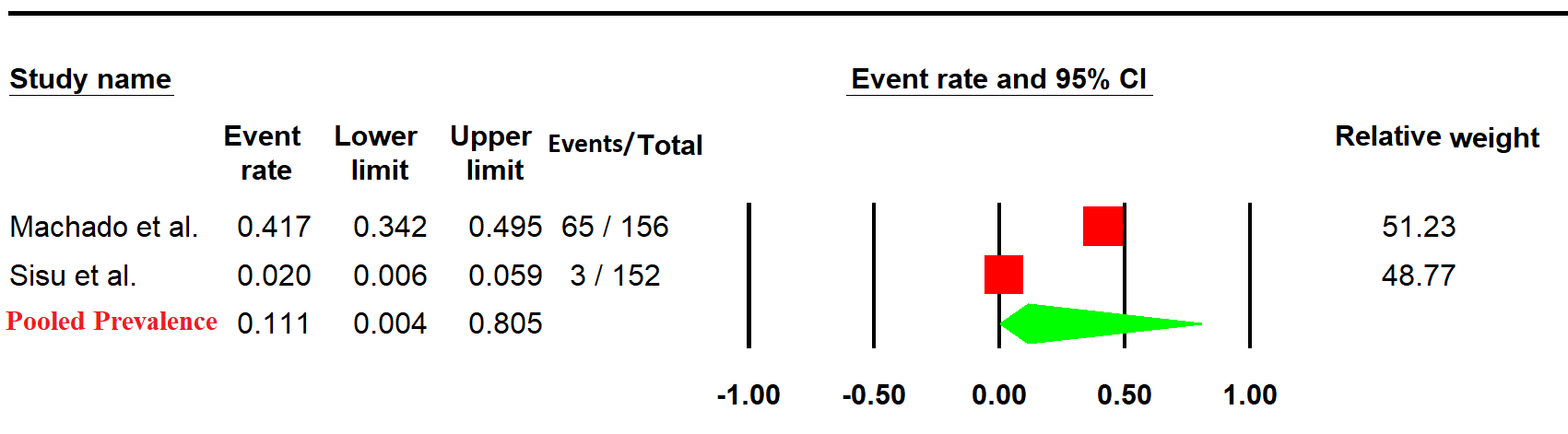


**Supplementary Figure 14.** Pooled prevalence of *Entamoeba coli* in cross-sectional studies.


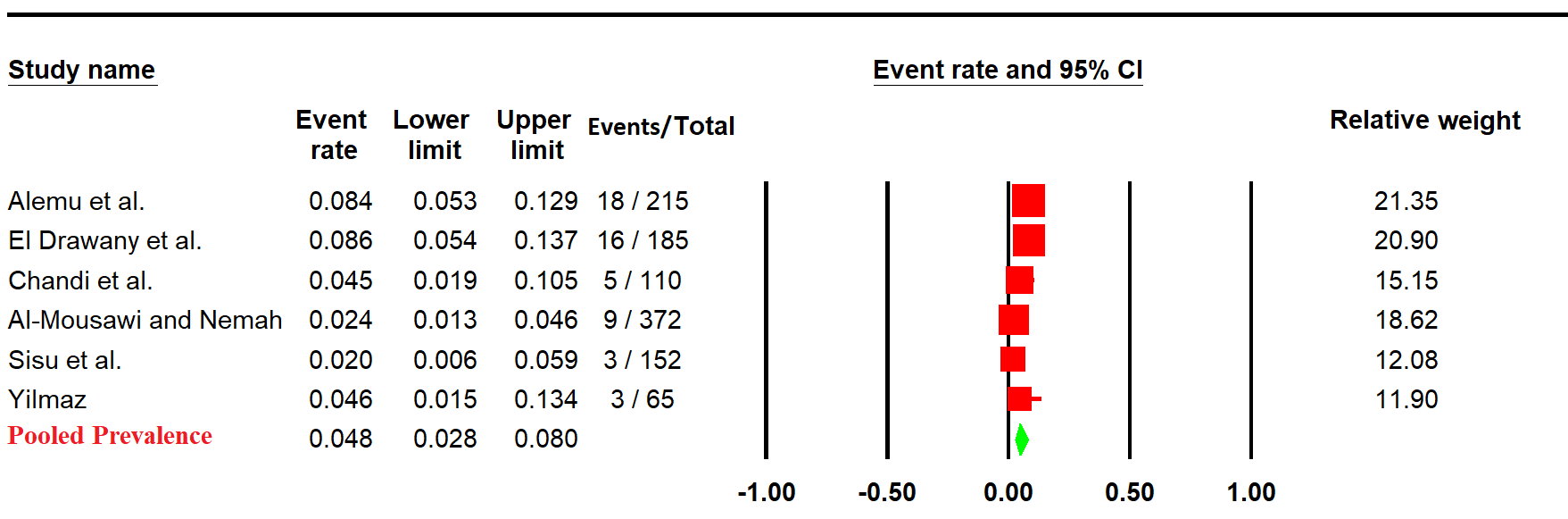


**Supplementary Figure 15.** Pooled prevalence of *Cryptosporidium* spp. in cross-sectional studies.


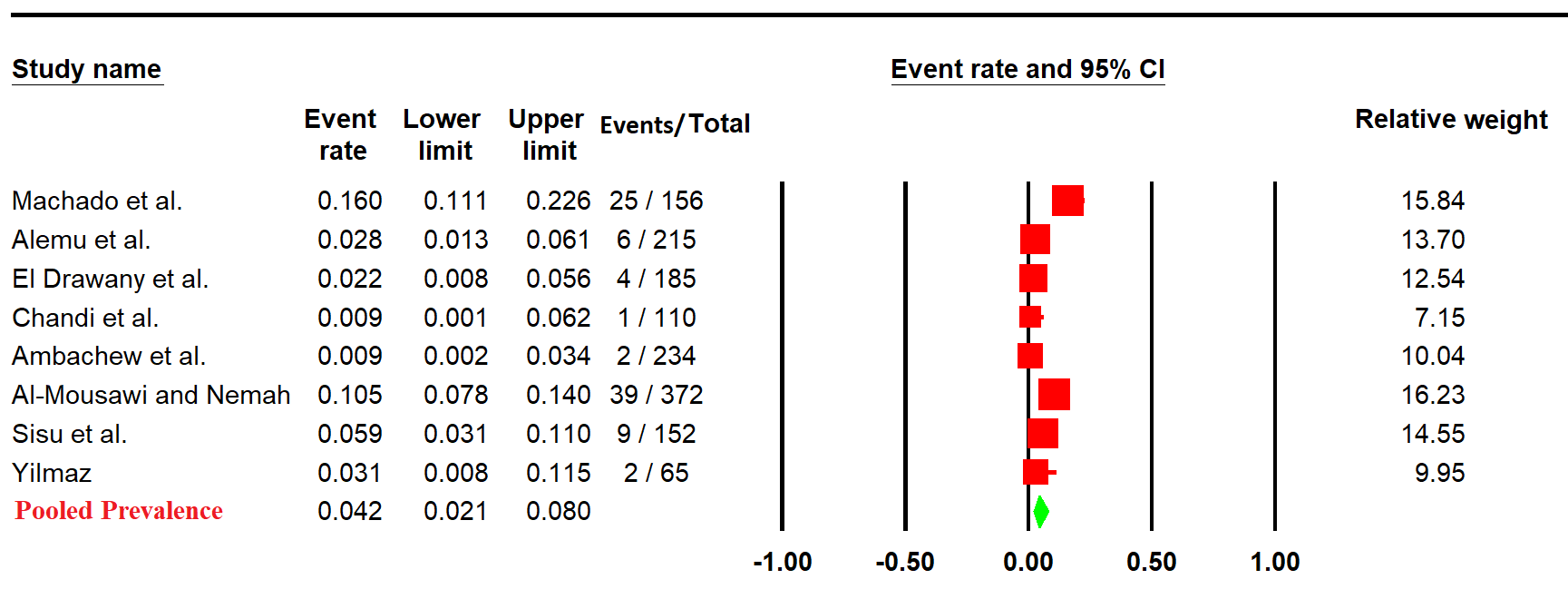


**Supplementary Figure 16.** Pooled prevalence of *Giardia* spp. in cross-sectional studies.


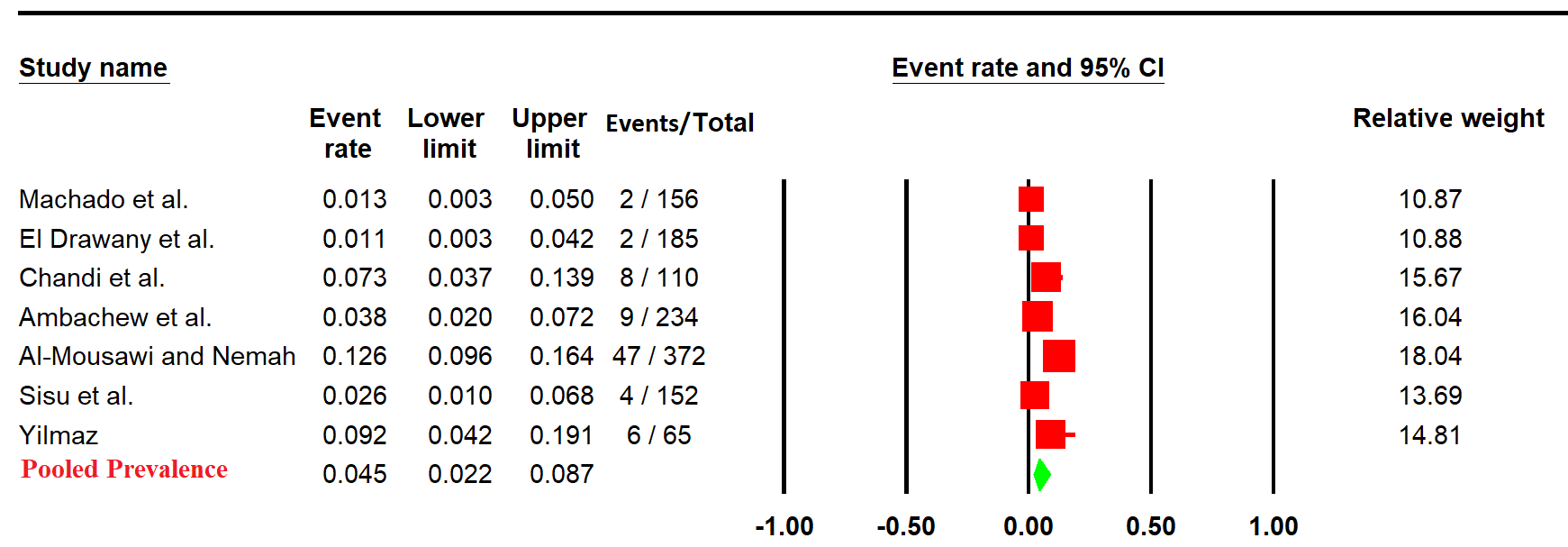


**Supplementary Figure 17.** Pooled prevalence of *Entamoeba histolytica/dispar* in cross-sectional studies.


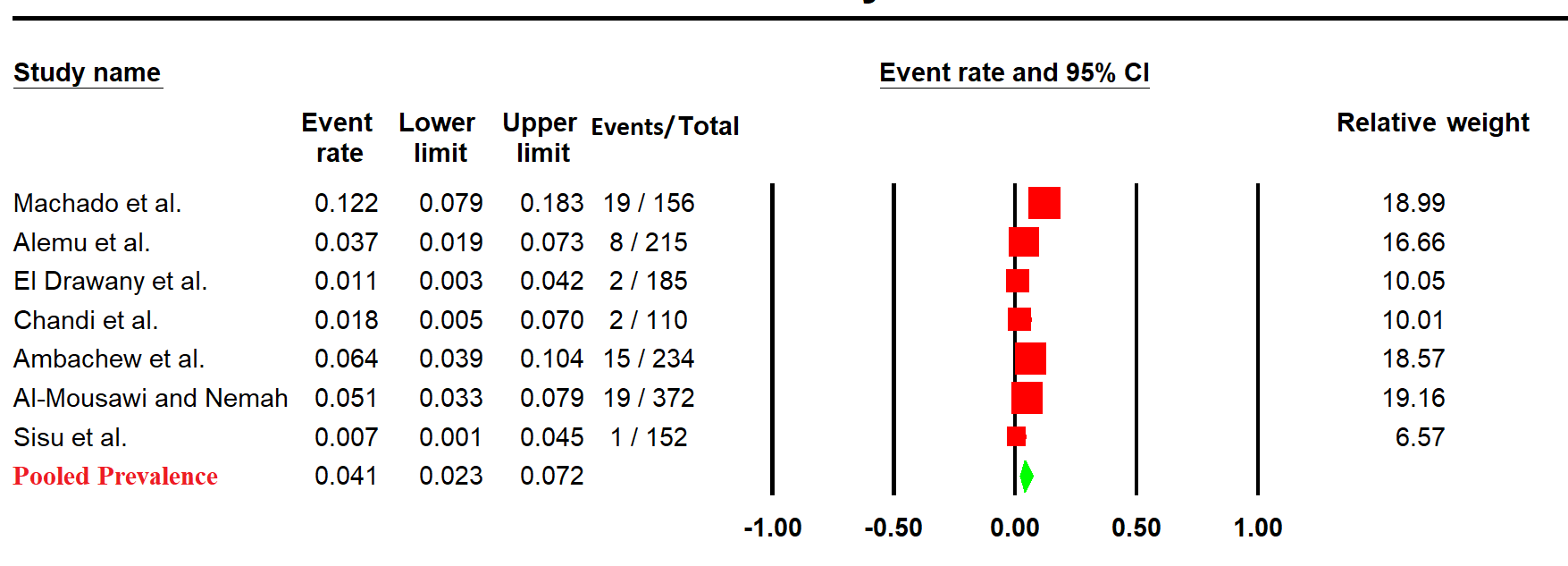


**Supplementary Figure 18.** Pooled prevalence of *Ascaris lumbricoides* in cross-sectional studies.


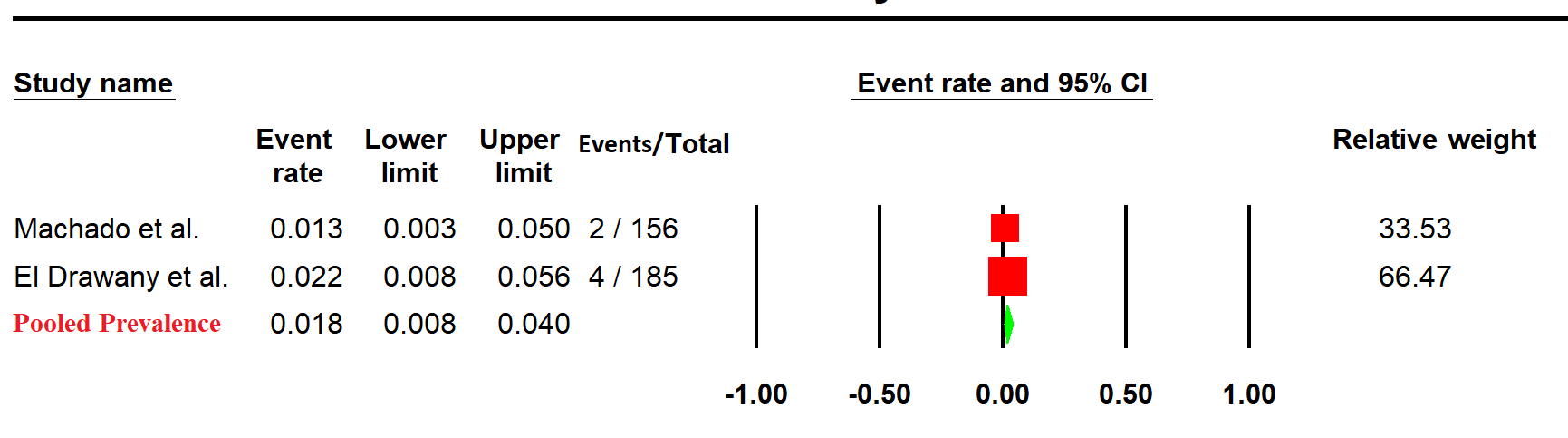


**Supplementary Figure 19.** Pooled prevalence of *Hymenolepis nana* in cross-sectional studies.


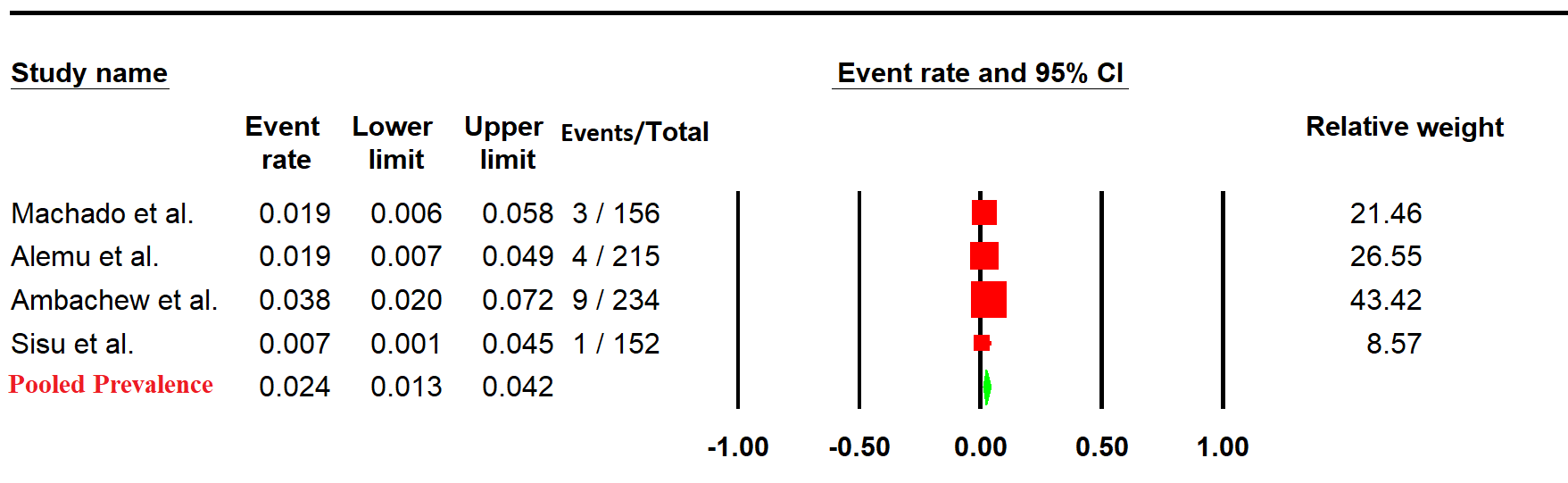


**Supplementary Figure 20.** Pooled prevalence of Hookworm in cross-sectional studies.


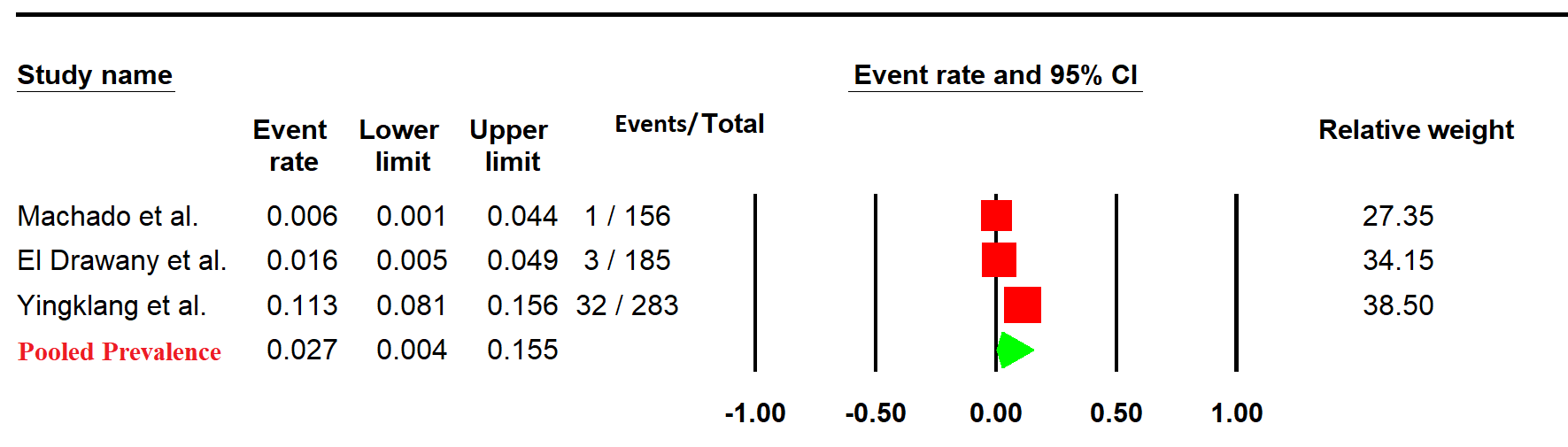


**Supplementary Figure 21.** Pooled prevalence of *Strongyloides stercoralis* in cross-sectional studies.
